# Cdc48 and its co-factor Ufd1 extract CENP-A from centromeric chromatin and can induce chromosome elimination in the fission yeast *Schizosaccharomyces pombe*

**DOI:** 10.1242/bio.060287

**Published:** 2024-04-08

**Authors:** Yukiko Nakase, Hiroaki Murakami, Michiko Suma, Kaho Nagano, Airi Wakuda, Teppei Kitagawa, Tomohiro Matsumoto

**Affiliations:** Radiation Biology Center, Graduate School of Biostudies, Kyoto University, Yoshida-Konoe cho, Sakyo ku, Kyoto 606-8501, Japan

**Keywords:** Cdc48, Chromosome, Cnp1, Ufd1, Centromere, Pombe

## Abstract

CENP-A determines the identity of the centromere. Because the position and size of the centromere and its number per chromosome must be maintained, the distribution of CENP-A is strictly regulated. In this study, we have aimed to understand mechanisms to regulate the distribution of CENP-A (Cnp1^SP^) in fission yeast. A mutant of the *ufd1^+^* gene (*ufd1-73*) encoding a cofactor of Cdc48 ATPase is sensitive to Cnp1 expressed at a high level and allows mislocalization of Cnp1. The level of Cnp1 in centromeric chromatin is increased in the *ufd1-73* mutant even when Cnp1 is expressed at a normal level. A preexisting mutant of the *cdc48^+^* gene (*cdc48-353*) phenocopies the *ufd1-73* mutant. We have also shown that Cdc48 and Ufd1 proteins interact physically with centromeric chromatin. Finally, Cdc48 ATPase with Ufd1 artificially recruited to the centromere of a mini-chromosome (*Ch16*) induce a loss of Cnp1 from *Ch16*, leading to an increased rate of chromosome loss. It appears that Cdc48 ATPase, together with its cofactor Ufd1 remove excess Cnp1 from chromatin, likely in a direct manner. This mechanism may play a role in centromere disassembly, a process to eliminate Cnp1 to inactivate the kinetochore function during development, differentiation, and stress response.

## Teaser

**Summary:** We found that Cdc48 ATPase and its cofactor Ufd1 remove excess Cnp1 from chromatin likely in a direct manner.

## INTRODUCTION

The centromere is a specialized chromosomal locus at which a microtubule attachment site termed kinetochore is assembled in mitosis. CENP-A is a variant of histone H3 preferentially found in centromeric chromatin and serves as a base of the kinetochore assembly (Black and Cleveland, 2011; Catania and Allshire, 2014; Quenet and Dalal, 2012). Because CENP-A determines the identity of the centromere, its distribution must be strictly regulated. The position and size of the centromere and its number per chromosome must be maintained for the faithful segregation of chromosomes. CENP-A distribution is likely regulated by two mechanisms, one to deposit CENP-A at the correct positions and another to remove excess CENP-A from chromatin.

Interestingly, accumulating evidence suggests that CENP-A is sometimes massively eliminated from the centromeric chromatin. In mouse NIH/3T3 cells treated with genotoxic reagents, CENP-A is eliminated from the centromeric chromatin and found at the periphery and inside the nucleolus. This process requires the ATM-mediated signaling pathway and chromatin remodeling factors. CENP-A elimination is considered a part of DNA damage response, which safeguards the genome (Hedouin et al., 2017). In a holocentric organism, *Ascaris suum*, a part of genomic DNA is eliminated in somatic precursor cells during early development. At regions that will be lost, CENP-A is dramatically diminished, resulting in the absence of kinetochores and nondisjunction of these regions in mitosis (Kang et al., 2016). Elimination of CENP-A also plays a vital role in the differentiation of *Arabidopsis thaliana*. Non-dividing pollen vegetative cells undergo loss of CENP-A dependently on the activity of Cdc48A proteins. The disappearance of CENP-A from the centromeres is accompanied by the decondensation of centromeric heterochromatin and activation of rRNA genes. Elimination of CENP-A is proposed to contribute to both the growth and prevention of cell division of the pollen cells (Merai et al., 2014).

Cdc48, also known as p97 or valosin-containing protein (VCP), is a member of the ATPase associated with diverse cellular activities (AAA) ATPase family (Halawani and Latterich, 2006; Woodman, 2003). Cdc48 is involved in important cellular processes, including endoplasmic reticulum-associated degradation (ERAD), spindle disassembly, membrane fusion, autophagy, and transcriptional control (Finley et al., 2012; Franz et al., 2016; Meyer et al., 2012; van den Boom and Meyer, 2018). It utilizes intrinsic ATPase activity, mediates the extraction of polyubiquitinated proteins from cellular compartments or protein complexes, and delivers them to the 26S proteasome for degradation (Raman et al., 2011; Verma et al., 2011). This function is referred to as “segregase”. The cellular function and localization of Cdc48/p97 are regulated by multiple cofactors, including the well-studied Ufd1-Npl4 heterodimer (Hanzelmann and Schindelin, 2017). Most cofactors are recruited to Cdc48/p97 via conserved binding domains, which aid in substrate recognition or processing by providing additional molecular properties. By combining ubiquitin-binding properties of Ufd1-Npl4 with the ATPase activity of Cdc48, Cdc48-Ufd1-Npl4 is thought to assist in extracting ubiquitinated proteins from higher-order complexes (Baek et al., 2013; Meyer et al., 2012). It has been recently discovered that Cdc48, Ufd1, and Npl4 are necessary for restricting CENP-A/Cse4 to centromeres in budding yeast. In these mutants, polyubiquitinated Cse4 accumulates in chromatin (Ohkuni et al., 2022).

We have previously reported that the Rpt3 protein, a subunit of the 19S proteasome, is involved in regulating the distribution of Cnp1 (Kitagawa et al., 2014). The *rpt3-1* mutant was identified through a screen for mutants that became temperature-sensitive upon overexpression of Cnp1. ChIP analysis revealed that Cnp1 was distributed broader and denser in the centromeric chromatin of the *rpt3-1* mutant, even when Cnp1 was expressed at a normal level.

In this study, we report that Cdc48 ATPase and its cofactor Ufd1 are required to maintain proper distribution of Cnp1 in fission yeast. In a mutant of the *ufd1^+^* gene, isolated from the screen the same as the *rpt3-1* mutant, the level of Cnp1 in centromeric chromatin is increased. Consistently, a mutant in the *cdc48*^+^ gene (*cdc48-353*) exhibits similar phenotypes. We have also found that both Cdc48 and Ufd1 proteins associate with the centromeric regions as well as other chromosomal regions. Finally, Cdc48 ATPase with Ufd1 artificially recruited to the centromere of a mini-chromosome (*Ch16*) induce a loss of Cnp1 from *Ch16*, resulting in an increased rate of chromosome loss. We propose that Cdc48 ATPase and its cofactor remove excess Cnp1 from chromatin, likely directly, to maintain proper distribution of Cnp1.

## RESULTS

### Centromeric chromatin in *ufd1-73* and *cdc48-353* mutants

**Fig. 1.**
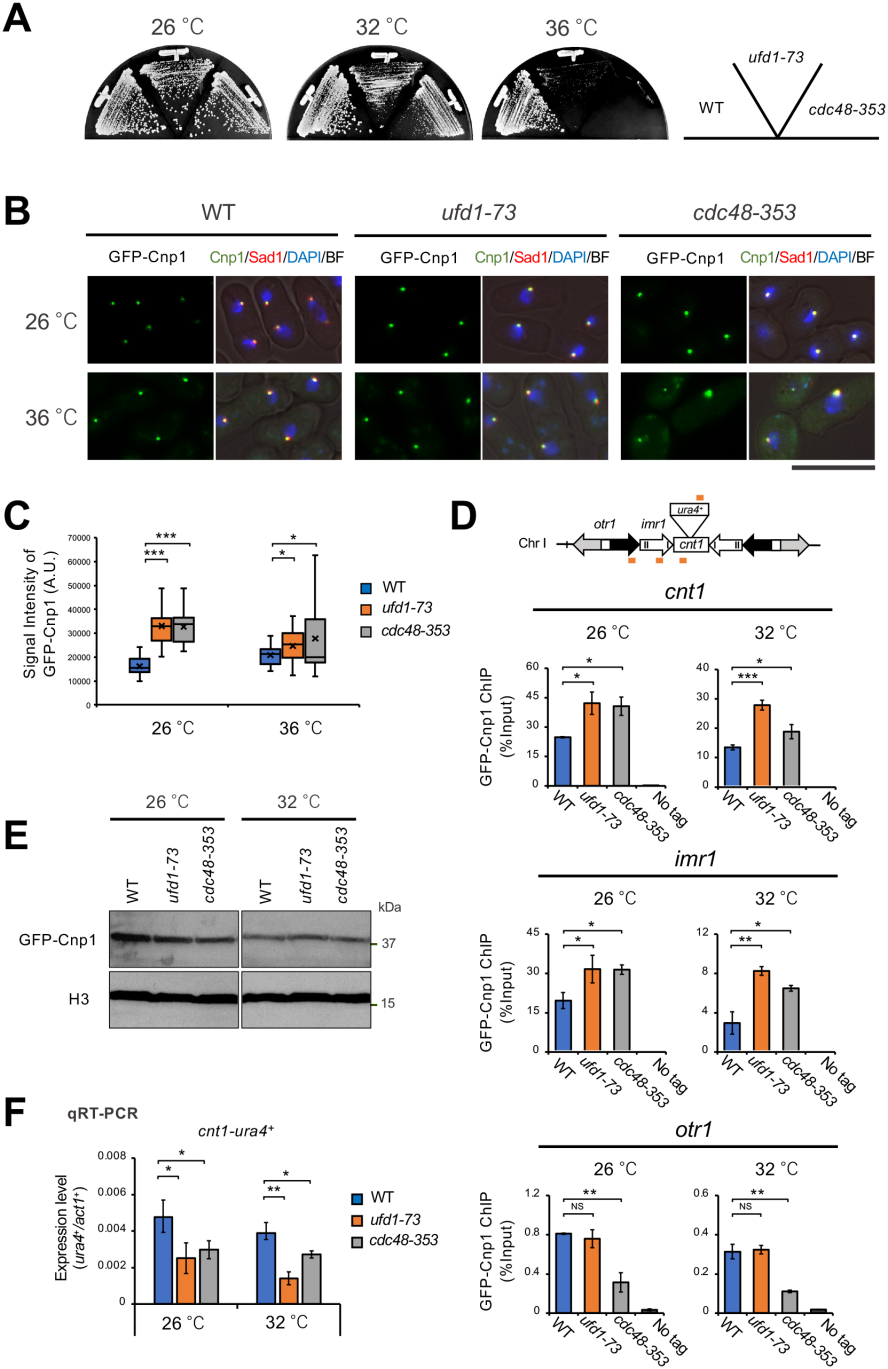
**Distribution of Cnp1 in *ufd1-73* and c*dc48-353*.** (A) Temperature sensitivity of *ufd1-73* and *cdc48-353* mutants. Strains were streaked on YEA plates. (B) Localization of GFP-Cnp1 from a native promoter. Strains expressing GFP-Cnp1 from native promoter were grown to mid-log phase in liquid YE medium at 26°C and shifted to 36°C for 6 h. Sad1-mCherry: SPB maker. Scale bar: 10 µm. (C) The statistical analysis of data represented in panel B. The GFP-Cnp1 intensity was calculated using NIH ImageJ and shown in arbitrary units (*n*=20). Representative experiments are shown (*n*=3). (D) Diagram showing localization of GFP-Cnp1 by ChIP analysis. Strains were grown to mid-log phase in liquid YE medium at 26°C and shifted to 32°C for 3 h. All data represent the mean±s.e.m. (*n*=3). The orange bars indicate the position of primers used for ChIP or qRT-PCR (F). (E) The protein level of GFP-Cnp1 detected by western blotting. Strains were grown to mid-log phase in liquid YE medium at 26°C and shifted to 32°C for 8 h. (F) Gene silencing of centromeric regions. Strains were grown as (D). qRT-PCR analyzed mRNA levels. Data are presented as mean±s.e.m. (*n*=3). *P*-values (unpaired *t*-test) comparing controls (WT): *P<0.05, **P<0.01, ***P<0.001, NS: not significant.

Our preliminary examination suggested that the *ufd1-73* and *cdc48-353* mutants could not remove excess Cnp1 from chromatin (Fig. S1). Because we attempted to investigate the role of Ufd1 and Cdc48 in the maintenance of proper distribution of Cnp1 in the centromeric chromatin under physiological conditions, the experiments described below were performed with cells expressing Cnp1 tagged with N-terminal fusion GFP from the native promoter (Kitagawa et al., 2014). When Cnp1 was solely expressed from its native promoter, the *ufd1-73,* and *cdc48-353* grew slightly slower than the wild-type strain at 32°C but could not grow at 36°C (Fig. 1A).

Microscopic observation of the mutants revealed that the signal from GFP-Cnp1 appeared as a single speckle but that the intensity of the fluorescent signal was about twice as strong as that of the wild-type cells at 26°C (Fig. 1B,C). However, the increase in signal intensity of GFP-Cnp1 at 36°C was smaller than that at 26°C. The reason for this may be due to an increase in the number of dead cells of the mutants, as shown in Fig. 1A. Since the growth of the mutants was observed at 32°C, the following experiments were performed with the limiting temperature of 32°C.

In fission yeast, each centromere contains a central domain (*cnt*) flanked by innermost repeats (*imr*), which provide the core of kinetochore formation. This core domain is surrounded by arrays of outer repeats (*otr*) (Partridge et al., 2000; Takahashi et al., 1992). Previous studies showed that Cnp1 is preferentially incorporated into *cnt* and *imr* but poorly into *otr* (Takahashi et al., 2000). ChIP analysis revealed that the amount of Cnp1 in *cnt* and *imr* chromatin of the mutants was increased (Fig. 1D). We also examined the level of GFP-Cnp1 by immunoblot with an antibody to GFP. As shown in Fig. 1E, the level of GFP-Cnp1 in the mutants was comparable to that of the wild-type cells. Gene silencing at the central domain (*cnt*) correlates with the amount of Cnp1 incorporated into the *cnt*-chromatin (Castillo et al., 2007). As shown in Fig. 1F and Fig. S2, the expression of the *ura4^+^* gene inserted at the *cnt* domain of *cen1* was silenced at 26°C and 32°C in the mutants.

The results described above indicated that while the *ufd1-73* and *cdc48-353* mutants expressed Cnp1 at a level similar to that of the wild-type strain, their centromeric chromatin at *cnt* and *imr* domains excessively incorporated Cnp1. Cdc48 ATPase and its cofactor, Ufd1, might be required to maintain proper distribution of Cnp1 at the centromere.

### Localization of Ufd1 and Cdc48

**Fig. 2.**
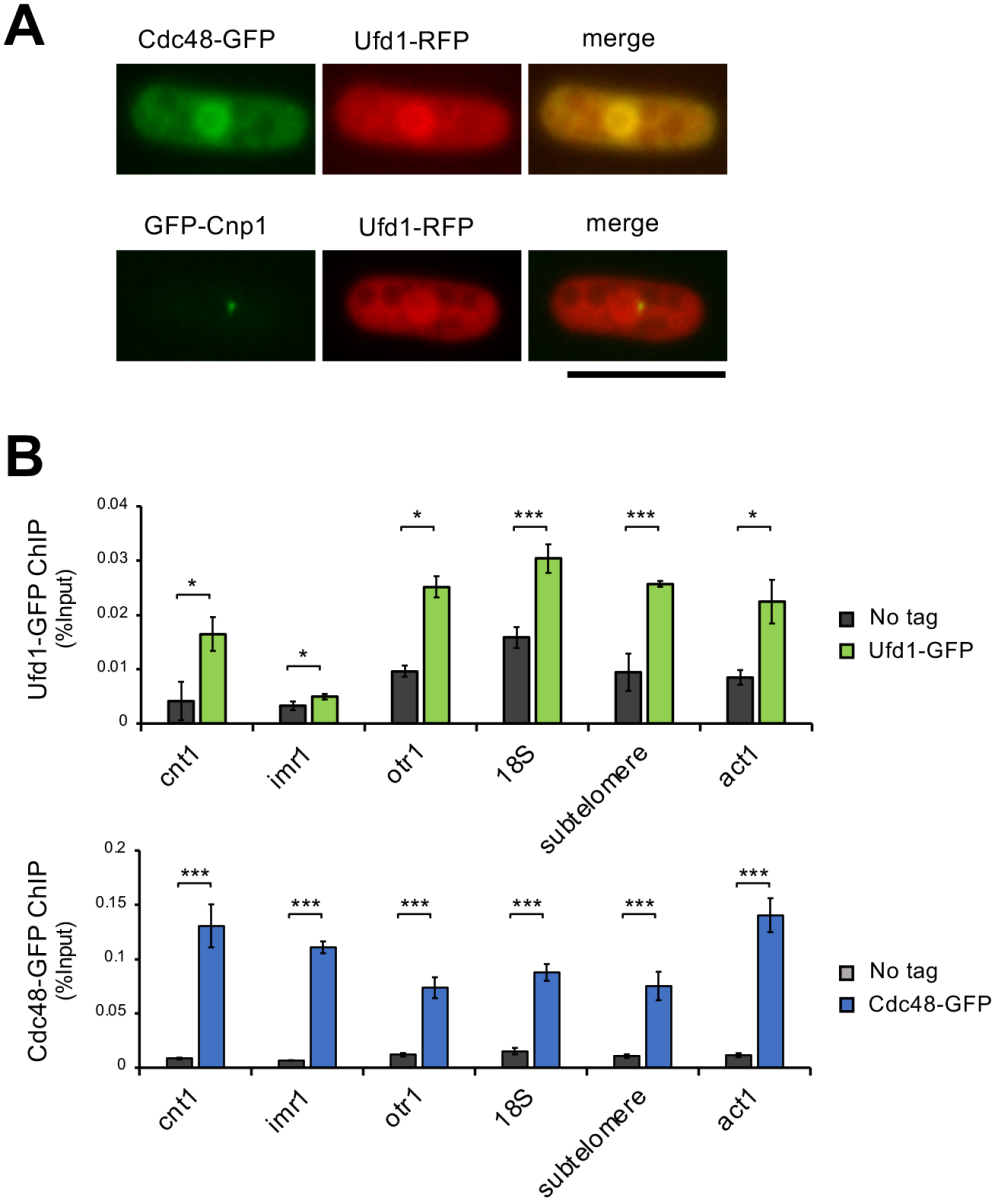
**Localization of Ufd1 and Cdc48.** (A) Localization of Ufd1-RFP, Cdc48-GFP, and GFP-Cnp1. Strains were grown to mid-log phase in liquid YE medium. Scale bar: 10 µm. (B) Localization of Ufd1-GFP and Cdc48-GFP by ChIP analysis. Strains were grown to mid-log phase in liquid YE medium at 26°C. All data represent the mean±s.e.m. (*n*=3). A two-sided unpaired *t*-test was used to calculate all *P*-values. *P<0.05, **P<0.01, ***P<0.001, NS: not significant.

A previous study demonstrated that the Cdc48 proteins were localized at chromatin and in the cytoplasm in fission yeast (Yuasa et al., 2004). We reinvestigated the localization of Cdc48 with Ufd1 and found that both GFP-tagged Cdc48 protein and RFP-tagged Ufd1 protein were localized in the chromatin domain as well as the cytoplasm (Fig. 2A). However, microscopic observation could not provide clear evidence for localization of the two proteins at centromeric chromatin. We, therefore, examined the association of Cdc48 and Ufd1 with centromeric chromatin more precisely by ChIP analysis. As shown in Fig. 2B, Cdc48 and Ufd1 proteins were found to localize to the centromeric regions as well as other chromosomal regions, *18S,* subtelomere, and *act1^+^*.

### Removal of Cnp1/CENP-A by Cdc48 segregase

**Fig. 3.**
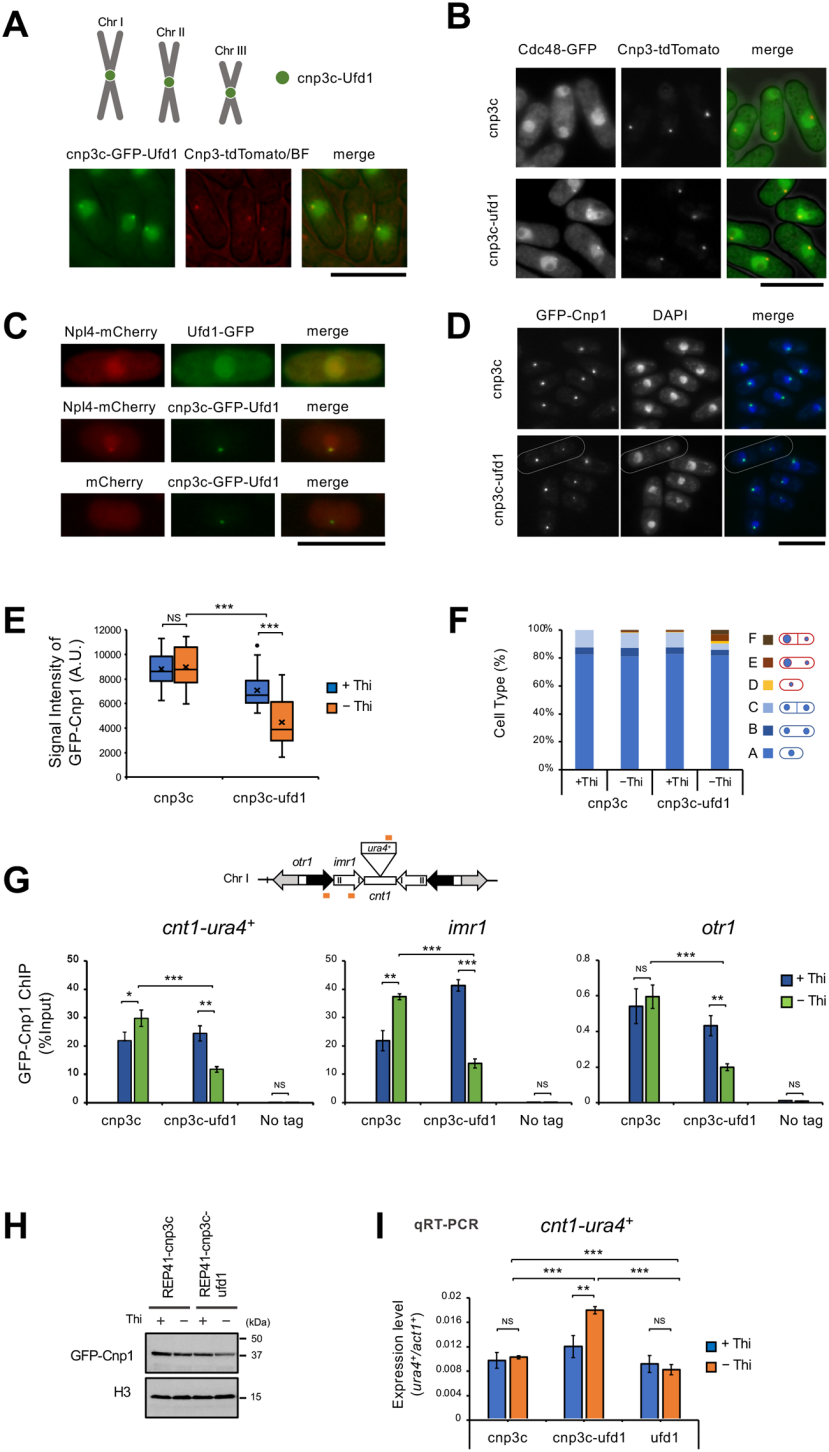
**Ufd1 inhibits the chromatin binding of Cnp1.** (A) Localization of cnp3c-GFP-Ufd1. The C-terminal domain (414-643 aa) of Cnp3 indicates centromere binding activity (Tanaka et al., 2009). We constructed the C-terminal domain of Cnp3 fused *ufd1^+^* plasmids. WT strains were transformed with pREP81-cnp3c-GFP-Ufd1. The resulting transformants were grown to mid-log phase in liquid EMM+thiamine medium at 26°C and then transferred to EMM-thiamine medium for induction for 24 h. (A-I) was observed under the same conditions. (A-D) scale bars: 10 µm. (B) Localization of Cdc48-GFP under expressing cnp3c-Ufd1. (C) Localization of Npl4-mCherry under expressing cnp3c-GFP-Ufd1. (D) Localization of GFP-Cnp1 under expressing cnp3c-Ufd1. White dotted lines indicate unevenly distributed cells. (E) The statistical analysis of the signal intensity of GFP-Cnp1 in panel D. (F) Cell types in (D). Cell type A-C: normal cells; D-F: abnormal cells. The GFP-Cnp1 intensity was calculated using NIH ImageJ and shown in arbitrary units (*n*=20). Representative experiments are shown (*n*=3). (G) Localization of GFP-Cnp1 by ChIP analysis under expressing cnp3c-Ufd1. All data represent the mean±s.e.m. (*n*=3). The orange bars indicate the position of primers used for ChIP. (H) The protein level of GFP-Cnp1 by detected western blotting under expressing cnp3c-Ufd1. (I) Gene silencing of centromeric regions under expressing cnp3c-Ufd1. qRT-PCR analyzed mRNA levels. Data are presented as mean±s.e.m. (*n*=3). A two-sided unpaired *t*-test was used to calculate all *P*-values. *P<0.05, **P<0.01, ***P<0.001, NS: not significant.

Cdc48 ATPase with Ufd1 acts as a ‘segregase’ that extracts proteins from cellular compartments or protein complexes (Baek et al., 2013; Meyer et al., 2012). Because our results described above indicated that Cdc48 ATPase and Ufd1 are associated with chromatin, and their dysfunction allows excess accumulation of Cnp1, we hypothesized that Cdc48 ATPase with Ufd1 extracts Cnp1 from chromatin. To test this hypothesis, we attempted to increase the level of Cdc48 segregase at centromeric chromatin and examine the amount of Cnp1. We ectopically expressed cnp3c-GFP-Ufd1, a fusion protein consisting of the C-terminal domain (414-643 aa) of Cnp3 previously shown to have a centromere binding activity (Tanaka et al., 2009), GFP as a visible marker, and Ufd1. As shown in Fig. 3A, the cnp3c-GFP-Ufd1 fusion protein was localized throughout the chromatin domain and concentrated at the centromeres. In separate experiments, we examined the cnp3c-Ufd1 fusion protein for its ability to recruit Cdc48 ATPase and Npl4, another cofactor functioning with Ufd1. As shown in Fig. 3B, Cdc48 ATPase tagged with GFP was concentrated at the centromeres when the cnp3c-Ufd1 fusion protein was ectopically expressed. Likewise, Npl4 tagged with mCherry was focused at the centromeres (Fig. 3C). Together, these results suggested that the three components (Cdc48, Ufd1, and Npl4) required for the functional Cdc48 segregase were recruited to centromeric chromatin by ectopic expression of the cnp3c-Ufd1 fusion protein. We next examined the three components for their ability to eliminate Cnp1. As shown in Fig. 3D and E, the fluorescent signal from GFP-Cnp1 in cells expressing the cnp3c-Ufd1 fusion protein was less than half that of cells expressing cnp3c alone. Although the expression level of Cnp1 was not changed (Fig. 3H), the reduction of Cnp1 in centromeric chromatin was confirmed by ChIP (Fig. 3G). Due to the decrease of Cnp1 at the centromeres, approximately 10% of the cells expressing the cnp3c-Ufd1 fusion protein exhibited abnormal chromosome segregation (Fig. 3F). Furthermore, gene silencing of the *ura4^+^* gene inserted at *cnt1* was loosened under expression of cnp3c-Ufd1 (Fig. 3I). In addition, we tested the expression of Cnp3c-GFP-Ufd1 in *cnp1-1* and *mis6-302* mutants, which have reduced centromere loading of Cnp1 under restrictive temperature, these mutants became more sensitive to high temperatures (Fig. S3). These results consistently indicated that the forced recruitment of Ufd1 to the centromeres attracted Cdc48 and Npl4, which then functioned as a segregase to extract Cnp1 from chromatin.

### Elimination of a mini-chromosome by Cdc48 segregase

We finally assessed the ability of the Cdc48 segregase to eliminate a specific chromosome by extracting Cnp1. Fission yeast mini-chromosome *Ch16* is a derivative of chromosome III, which lacks most of the arm regions but maintains the centromere III (Niwa et al., 1989). It is a model chromosome transmitted stably and is not essential for viability.

**Fig. 4.**
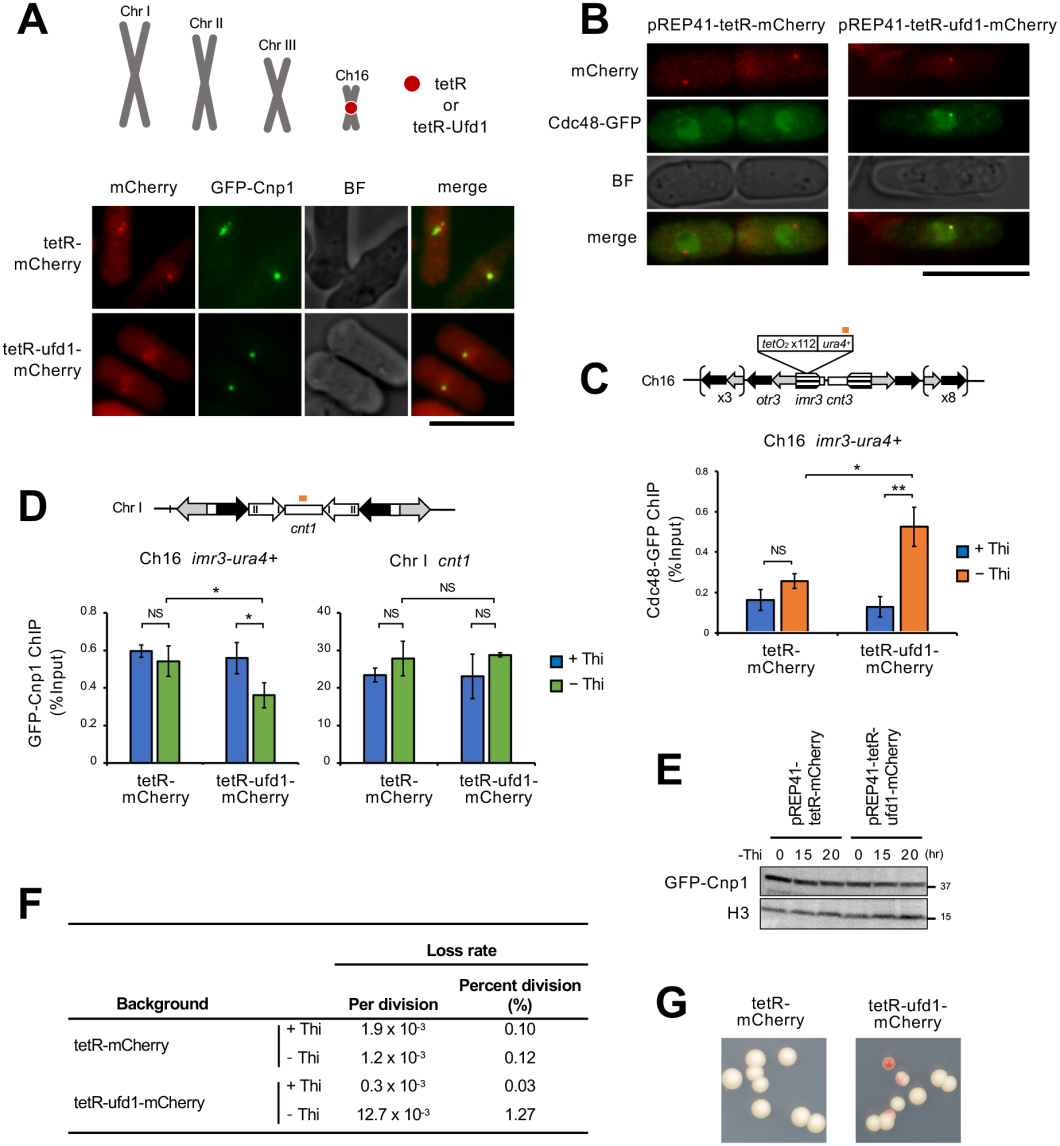
**Artificial enrichment of Ufd1 at the centromere causes chromosome instability.** (A) Artificial enrichment of Ufd1 at the centromere of mini-chromosome *Ch16* using tetO/tetR system. We constructed tetR fused *ufd1^+^* plasmids. The strains containing *tetO* sequence inserted centromeric region were transformed with pREP41-tetR-mCherry or pREP41-tetR-Ufd1-mCherry. The resulting transformants were grown to mid-log phase in liquid EMM+thiamine medium at 30°C and then transferred to EMM-thiamine medium for induction for 24 h. (A-D) was observed under the same conditions. Scale bars: 10 µm. (B) Localization of Cdc48-GFP under expressing tetR-Ufd1-mCherry. (C) Localization of Cdc48-GFP by ChIP analysis under expressing tetR-Ufd1-mCherry. All data represent the mean±s.e.m. (*n*=3). The orange bar indicates the position of the primer used for ChIP. (D) Localization of GFP-Cnp1 by ChIP analysis under expressing tetR-Ufd1-mCherry. All data represent the mean±s.e.m. (*n*=3). The orange bar indicates the position of the *cnt1* primer used for ChIP. The primer of *imr3-ura4^+^* used for ChIP was the same as (C). (E) The protein level of GFP-Cnp1 by western blot under expression terR-Ufd1-mCherry. The expression level of Cnp1 was not changed. Ufd1-mCherry does not affect the expression level of Cnp1. (F) Stability of mini-chromosome. The stability of *Ch16* was determined on YE-Ade media at 30°C. (G) The colonies on YE-Ade under expressing tetR-mCherry or tetR-Ufd1-mCherry. A two-sided unpaired *t*-test was used to calculate all *P*-values. *P<0.05, **P<0.01, NS: not significant.

A plasmid expressing tetR-Ufd1-mcherry, a fusion protein consisting of tetR, Ufd1, and mCherry as a visible marker, was transformed into a strain in which *tetO* array with *ura4^+^* cassette was inserted at the *imr3 L* domain of *Ch16.* We anticipated that Ufd1 could be recruited to the *imr3 L* domain by tetR, which recognized *tetO* arrays. As shown in Fig. 4A, upon induction of the fusion protein, tetR-Ufd1-mCherry, the fluorescent signal from mCherry appeared as a single speckle and was colocalized with GFP-Cnp1, suggesting that Ufd1 was recruited to the centromere of *Ch16* as anticipated. The tetR-Ufd1-mCherry fusion protein could also recruit Cdc48-GFP to the centromeric region of *Ch16* (Fig. 4B and C). Based on these results, we speculated that the functional Cdc48 segregase was recruited to the centromere of *Ch16*. We then examined the level of Cnp1 at the centromere of *Ch16*. As shown in Fig. 4D and E, induction of the tetR-Ufd1-mCherry fusion protein significantly decreased the level of Cnp1 at the centromere of *Ch16,* but did not affect the centromere of Chromosome I. We next investigated whether reduced levels of Cnp1 affect the function of the *Ch16* centromere. We measured the rate of *Ch16* loss per division, which is indicated by red rather than white colonies on a limited adenine plate. By calculating the frequency of red colonies, we accurately determined the rate of chromosome loss per division. Our findings revealed that the rate of *Ch16* loss per division was about 10-fold higher than in cells expressing tetR-mCherry, a fusion protein lacking Ufd1 (Fig. 4F and G). These results suggested that the Cdc48 segregase disassembled the centromeric chromatin and induced the elimination of *Ch16*.

## DISCUSSION

In this study, *S. pombe* Cdc48 and its cofactor Ufd1 have been characterized with a focus on their role at the centromeric chromatin. We have shown by performing ChIP that a subset of each of the two proteins is localized at the centromeric chromatin. In the corresponding mutants (*cdc48-353* and *ufd1-73*), CENP-A (fission yeast Cnp1) excessively accumulates in the centromeric chromatin at the *cnt* and *imr* but not at the *otr* domains. These two lines of evidence suggest that Cdc48, along with Ufd1, play an important role in the maintenance of proper distribution of Cnp1 at the core of the centromeric chromatin. It has been reported in a variety of organisms that the Cdc48-Ufd1-Nlp4 complex removes specific proteins from chromatin (Dobrynin et al., 2011; Hu et al., 2004; Kohler et al., 2013, 2015; Ramadan et al., 2007; Sasagawa et al., 2012; Singh et al., 2020). We propose that the Cdc48 complex targets Cnp1 as well and removes excess Cnp1 from centromeric chromatin. The function of Cdc48 with its co-factors as the Cnp1 segregase is likely conserved through evolution.

We have previously reported that the 26S proteasome (or 19S proteasome subcomplex) associates with the centromeric chromatin and regulates the distribution of Cnp1 (Kitagawa et al., 2014). The mutant of the gene encoding Rpt3, a subunit of the 19S subcomplex, was prone to excessive accumulation of Cnp1 in centromeric chromatin. However, the amount of intracellular Cnp1 was comparable to that of the wild-type strain. This suggests that the excessive incorporation of Cnp1 into centromeric chromatin is not due to a defect in Cnp1 degradation. *cdc48-353* and *ufd1-73* mutants also showed no difference in intracellular Cnp1 levels from the wild-type. In budding yeast, the accumulation of polyubiquitinated Cse4 in the Cdc48 complex mutants indicates that Cse4 removed by the Cdc48 complex is degraded by the proteasome (Ohkuni et al., 2022). We hypothesize that the amount of Cnp1 incorporated into centromeric chromatin in fission yeast is limited by another mechanism rather than by the degradation seen in budding yeast. Interestingly, the *otr* domain of *rpt3-1* is more prone to excessive accumulation of Cnp1 than the other two domains, *cnt,* and *imr*, while the *cdc48-353* and *ufd1-73* mutants, conversely, are prone to Cnp1 accumulation in the *cnt* and *imr* domains. These observations suggest that the 26S proteasome (or 19S proteasome subcomplex) and Cdc48 with Ufd1 regulate the level of Cnp1 at the centromeric domain independently.

To demonstrate more directly that the Cdc48 complex serves as the Cnp1 segregase, we recruited the Cdc48 complex to the centromeric chromatin and monitored the level of Cnp1. When the Cdc48 complex was recruited to the centromeric chromatin of the regular chromosomes, the level of Cnp1 at the *cnt* domain of chromosome I decreased to approximately 50%. Likely due to the loss of Cnp1 from the centromeric chromatin, some chromosomes lost the centromeric activity and segregated abnormally. Likewise, when the Cdc48 complex was recruited specifically to an artificial chromosome, *Ch16*, the level of Cnp1 at the *imr* domain of *Ch16* decreased by approximately 30%, and the loss rate of *Ch16* increased by 10-fold. These results suggest that the Cdc48 complex is a Cnp1 segregase. There are reports that the incorporation of Cnp1 into the centromere region is promoted by transcription (Singh et al., 2020). RNA polymerase II (RNAPII) accumulates in fission yeast centromeres, suggesting that transcription-coupled chromatin remodeling events may promote H3 elimination and replacement of H3 by Cnp1. Since budding yeast Cdc48 is also involved in RNAPII turnover (Verma et al., 2011), it is possible that fission yeast Cdc48 also regulates Cnp1 in the centromere region via RNAPII turnover.

How does Cdc48 recognize excess amounts of Cnp1? Cse4 in *S. cerevisiae* is ubiquitinated, and CenH3 in *A. thaliana* is sumoylated, and the Cdc48 complex recognizes those modified Cse4/CenH3 (Merai et al., 2014; Ohkuni et al., 2022). Since Cnp1 in *S. pombe* has also been reported to be ubiquitinated, albeit in an overexpression system (Yang et al., 2018), it is expected that the Cdc48 complex in fission yeast would also recognize ubiquitinated Cnp1.

## MATERIAL AND METHODS

### Yeast strains and growth media

The *S. pombe* strains used in this study are listed in Table S1. As described previously, *S. pombe* cells were grown in YEA and EMM containing the appropriate nutrient supplements (Moreno et al., 1991). All yeast transformations were performed with the lithium acetate method (Gietz et al., 1992; Okazaki et al., 1990).

### Plasmid construction

The plasmid used for overexpression studies is a derivative of pREP81 and pREP41 vectors with a thiamine-repressible promoter *nmt1^+^*. The expression level varies depending on the strength of the promoter, with pREP41 being stronger than pREP81. Plasmid pREP81-cnp3c-GFP-ufd1 was constructed as follows. The Gibson assembly inserted the ufd1 cDNA into the plasmid which the C-terminus 414-643 aa of *cnp3* (0.8 kb) and GFP (0.7 kb) are linked downstream of the nmt81 promoter.

Plasmid pREP41-cnp3c-ufd1 was constructed as follows. The Gibson assembly inserted the ufd1 cDNA (-1 kb) into the plasmid, which the C-terminus 414-643 aa of *cnp3* are linked downstream of the nmt41 promoter.

Plasmid pREP41-tetR-ufd1-mCherry was constructed as follows. A 0.6 kb *Sal*I(*BamH*I)-*Not*I fragment containing tetR was cloned into the corresponding site of pREP41-mCherry. A 1 kb *BamH*I-*Not*I fragment of *ufd1* cDNA was cloned into the corresponding site of pREP41-tetR-mCherry.

### Isolation of the *ufd1-73* mutant

A strain (*h^-^ leu1-32*::*nmt1-GFP-cnp1^+^-leu1^+^*) that was able to express fission yeast CENP-A, Cnp1, tagged with GFP from the nmt1 promoter, was chemically mutagenized as described (Matsumoto and Beach, 1991). Exponentially growing cells suspended in TM buffer [50 mM Tris malate (pH 6.0)] were treated with NTG (500 µg/ml) at 26°C for 20 min and washed three times with TM buffer. Cells were grown in the YES liquid medium for 4 h at 26°C and washed with EMM medium containing thiamine. Cells were grown in the same medium and grown at 26°C. The survivors were plated on the EMM plates containing thiamine and grown at 26°C. They were subsequently transferred onto EMM plates with and without thiamine by replica and grown at 36°C. The strains that exhibited temperature sensitivity only when GFP-Cnp1 was expressed were selected. They were then individually examined under a fluorescence microscope. We scored mutants with abnormal distribution of GFP-Cnp1 in the nucleus. Tetrad analysis indicated that five strains, including the *ufd1-73* mutant, carried a single mutation responsible for the phenotypes (temperature sensitivity and abnormal distribution of GFP-Cnp1).

### Isolation of the *ufd1^+^* gene

A genomic DNA fragment that complements the temperature sensitivity was isolated from a fission yeast genomic DNA library using temperature sensitivity as a selection marker. The integration mapping proved that this fragment originated from the *ufd1-73* locus. A PCR-based strategy was used to identify the mutation site within the *ufd1^+^* coding sequence. The *ufd1-73* mutant gene contained a single point mutation changing GGT (glycine) of the 55th codon to GAT (aspartic acid).

### Western blotting

Cell extracts were prepared from exponentially growing *S. pombe*. The cells were suspended in lysis buffer (25 mM HEPES-KOH at pH 7.5, containing 200 mM NaCl, 10% glycerol, 0.2% NP-40, and protease inhibitors (Nacalai Tesque), 1 mM PMSF, and 1% Trasylol) were disrupted by Beads Smash 12 (WAKENYAKU) at 4°C. After centrifugation, the supernatants were used for SDS-polyacrylamide gel electrophoresis (SDS-PAGE). Polypeptides were resolved by SDS-PAGE gel and then transferred onto nitrocellulose membranes (Funakoshi). Antibodies for western blotting were diluted as follows: mouse anti-GFP (Roche, 45-11814460001) 1:1000; rabbit anti-H3 (Abcam, ab1791) 1:1000. Blots were developed using ECL reagents (ThermoFisher Scientific).

### Microscopy

Two methods acquired images. One method was with a DM5500B fluorescent microscopy (Leica MICROSYSTEMS, Wetzlar, Germany) equipped with a HAMAMATSU ORCA-ER DIGITAL CAMERA (Hamamatsu Photonics, Hamamatsu, Japan) and processed with IPLab/Win (SOLUTION SYSTEMS, Funabashi, Japan). The other method was acquired with an all-in-one fluorescence microscope (BZ-8000, KEYENCE, Osaka, Japan) equipped with a Plan Apochromat 100× objective and processed with BZ-X Analyzer software (BZ-H4A, KEYENCE, Osaka, Japan).

### Fluorescent intensity of GFP-Cnp1

Fluorescent intensity was quantified with ImageJ 1.53a (National Institutes of Health, USA). The obtained data were represented in box-and-whisker plots. The box represents the interquartile range (IQR), encompassing the middle 50% of the data. It extends from the first quartile (Q1) to the third quartile (Q3), with the median (Q2) indicated by the line inside the box. The X in the box indicates the median, the data set's central value. The error bars extend from the box's ends to indicate the data's range, excluding outliers.

### ChIP assay

Exponentially growing cells (1×10^8^ cells) were fixed with 1% formaldehyde for 60 min. The reaction was then quenched by adding 330 mM final concentration of glycine to the culture and incubating for 5 min. The cells were collected by centrifugation and washed three times with PBS. Cell lysates were prepared by glass beads vortexing in FA-lysis buffer [50 mM HEPES-KOH (pH7.5), 150 mM NaCl, 1 mM EDTA, 0.1% sodium deoxycholate, protease inhibitor cocktail (Sigma-Aldrich, p8215), 1 mM PMSF] containing 0.5% SDS. After discarding the glass beads, the cross-linked chromatin was pelleted by centrifugation at 12,000 ***g*** for 20 min at 4°C, washed once with FA-lysis buffer containing 0.1% SDS. After that, the pellet was resuspended in the same buffer and sonicated using Bioruptor (Cosmo Bio) at 4°C to yield fragments with the average size of 500 bp. The sonicated sample was clarified by centrifugation at 12,000 ***g*** for 20 min at 4°C. The resultant soluble chromatin solution was supplemented with 1% Triton X-100. ChIP assay was performed by applying mouse anti-GFP (Roche) to the chromatin solution for 2 h at 4°C, followed by overnight incubation with Protein G-conjugated Dynabeads (Invitrogen) at 4°C. The beads were washed three times in FA-lysis buffer containing 0.1% SDS and 1% Triton X-100; once in the same buffer containing 0.5 M NaCl; once in 10 mM Tris-HCl (pH8.0), 250 mM LiCl, 1 mM EDTA, 0.5% NP-40 and 0.5% sodium deoxycholate; once in TE. To elute DNA and revert cross-linking, the beads were incubated with SDS-TE (TE containing 1% SDS) for 15 h at 65°C, followed by digestion with proteinase K (Merck) for 2 h at 42°C. After extraction with phenol and phenol-chloroform-isoamyl alcohol, DNA was precipitated with isopropanol in the presence of glycogen and suspended in TE. ChIP analysis was performed by quantifying the amount of DNA in ChIP and whole extract samples (WCE) using MyiQ2 (Bio-rad). ChIP DNA amount relative to WCE was determined by calculating their Ct value difference. The nucleotide sequences of the primer sets used in this study are listed in Table S2.

### qRT-PCR

Total RNAs were prepared from cultured *S. pombe* as described previously (Jensen et al., 1983). Aliquots of total RNA were used for the synthesis of cDNA by the ReverTra Ace quantitative real-time RT-PCR (qRT-PCR) Master Mix with gDNA Remover (TOYOBO) in accordance with the manufacturer's instructions. The synthesized cDNAs were quantified using MyiQ2 (Bio-Rad) and SYBR qPCR Mix (Bio-Rad). Expression profiles for *ura4^+^* were normalized against *act1*^+^ cDNA levels. The *ura4* primer sets are located in the region (0.2 kb) lacking in *ura4-DS/E* (Table S2).

### Mini-chromosome stability assay

The stability of the mini-chromosome was determined by methods by Allshire et al., 1995 and Suma et al., 2018. First, the cells were plated on YE or EMM containing 10 μg/ml adenine to determine the initial percentage of the cells carrying the mini-chromosome. After incubation in the non-selective medium (Ade^+^), the final percentage of the cells carrying the mini-chromosome was determined. The rate of mini-chromosome loss per division was estimated using the formula: loss rate=1-(F/I)^1/N^ (F, the final percentage of cells carrying mini-chromosome; I, the initial percentage of cells carrying mini-chromosome; N, the number of generations between F and I).

## Supplementary Material

10.1242/biolopen.060287_sup1 Supplementary information
