# Supplementary material for: Cdc48 and its co-factor Ufd1 extract CENP-A from centromeric chromatin and can induce chromosome elimination in the fission yeast Schizosaccharomyces pombe
Source: Biol Open. 2024 Apr 8;13(4):bio060287. doi: 10.1242/bio.060287 (PMC11033524; doi:10.1242/bio.060287)
Supplement: Supplementary information [file biolopen-13-060287-s1.pdf]

Supplementary Materials and Methods

Yeast strains and growth media

The *S. pombe* strains used in this study are listed in Supplementary Table 1. *S. pombe* cells were grown in YEA and EMM containing the appropriate nutrient supplements as described previously (Moreno et al., 1991). All yeast transformations were performed with the lithium acetate method (Gietz et al., 1992; Okazaki et al., 1990).

Western blotting

Crude cell extracts were prepared from *S. pombe* as described previously (Masai et al., 1995). Polypeptides were resolved by SDS-PAGE gel and then transferred onto nitrocellulose membranes. Antibodies for western blotting were diluted as follows: mouse anti-GFP (Roche) 1:1000; rabbit anti-H3 (1729, abcam) 1:1000. Blots were developed using ECL reagents (Thermo).

Microscopy

Images were acquired on a LEICA DM5500B (Leica) microscope equipped with a HAMAMATSU ORCA-ER camera and a KEYENCE (BZ-8000).

ChIP assay

ChIP was performed as described previously (Kitagawa et al., 2014). The nucleotide sequences of the primer sets used in this study are listed in Supplementary Table 2.

Biology Open • Supplementary information

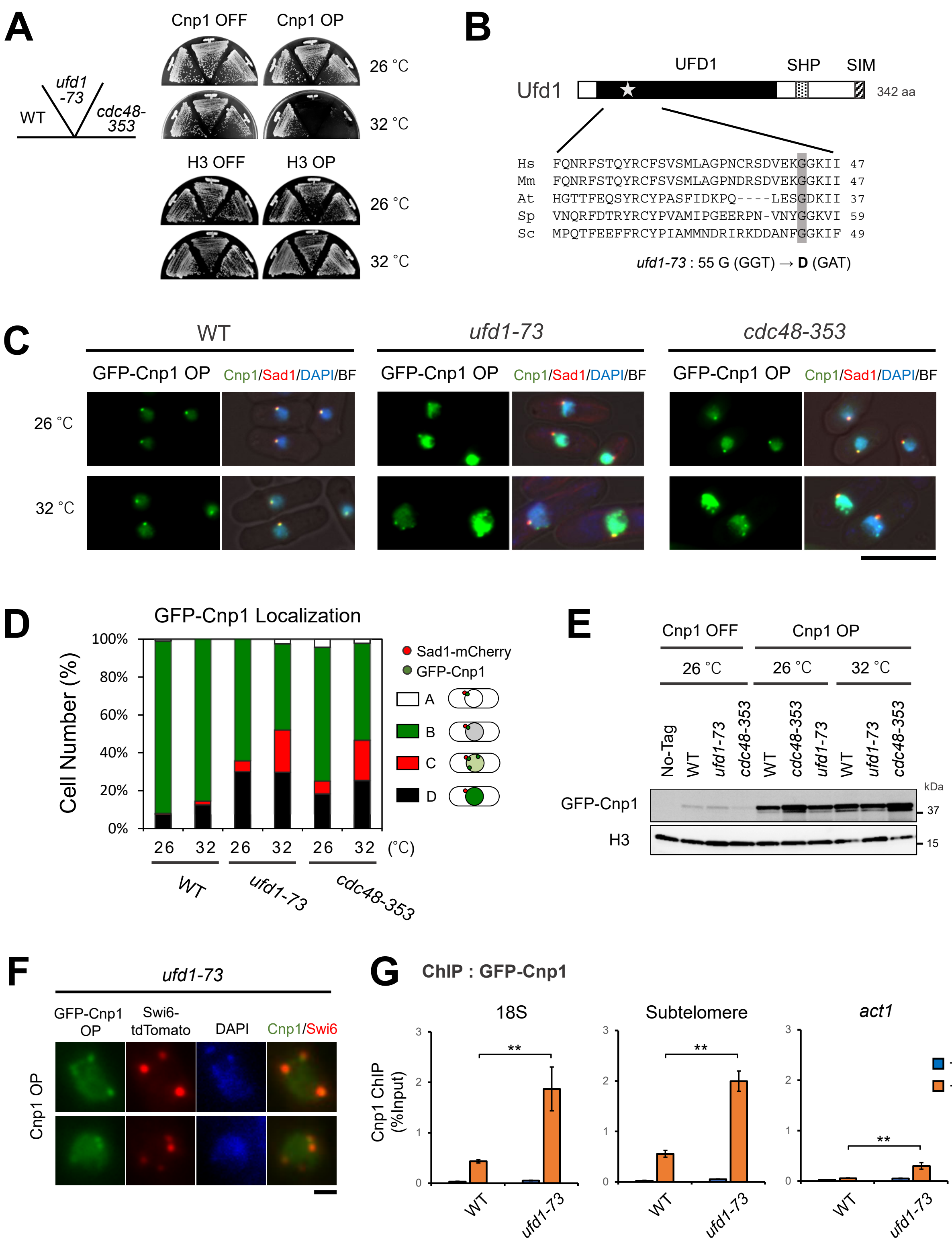

Fig. S1. *ufd1-73* mutants are sensitive to over-expression of CENP-A/Cnp1.
(A) Effect of over-expression of Cnp1 and histone H3. Strains ectopically expressing Cnp1 or histone H3 from nmt1 promoter were plated on EMM plates with thiamine for repression (OFF) or without thiamine for derepression (ON) at 26 ℃ or 32 ℃. (B) Schematic illustration of the structure of Ufd1 protein and the position of the *ufd1-73* mutation. Partial amino-acid sequences of Ufd1 from five species are aligned. The mutation site in fission yeast Ufd1 is a single point mutation changing GGT (glycine) of the 55th codon to GAT (aspartic acid). UFD1: ufd1 domain; SHP: p97 N domain binding and SIM: SUMO-interaction motif. (C) Localization of over-expressing GFP-Cnp1. Strains were grown to mid-log phase in liquid EMM+thiamine medium at 26 ℃ and then transferred to EMM-thiamine medium for induction of GFP-Cnp1 in absence of thiamine. After the induction of GFP-Cnp1 for 18 hr, they were shifted to 32 ℃ for 8 hr. Sad1-mCherry: SPB maker. scale bar: 10 μm. (C-E) were observed under same conditions. (D) The statistic analysis of (C). (E) The protein level of over-expressing GFP-Cnp1 by western blot. (F) Localization of Swi6 in *ufd1-73* under over-expressing Cnp1. Strains were grown as (C). scale bar: 2 μm. (G) Localization of over-expressing GFP-Cnp1 by ChIP analysis. Strains were grown as (C). All data represent the mean±s.e.m. (n=3). P-values (unpaired t-test) comparing controls (WT): **p<0.01.

Biology Open • Supplementary information

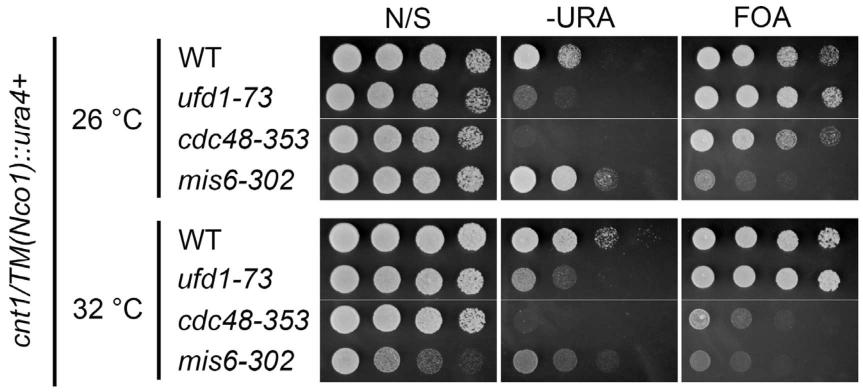

Fig. S2. Silencing of *cnt1::ura4⁺* is enhanced in *ufd1-73* and *cdc48-353* mutants.

The reporter gene *ura4⁺* was integrated at *cnt1* and examined its expression in each strain. The *mis6-302* temperature-sensitive mutant is known to be impaired in *cnt1* silencing (Pidoux et al., 2003). Consistent with Fig.1F, silencing of *cnt1::ura4⁺* was enhanced in *ufd1-73* and *cdc48-353*.

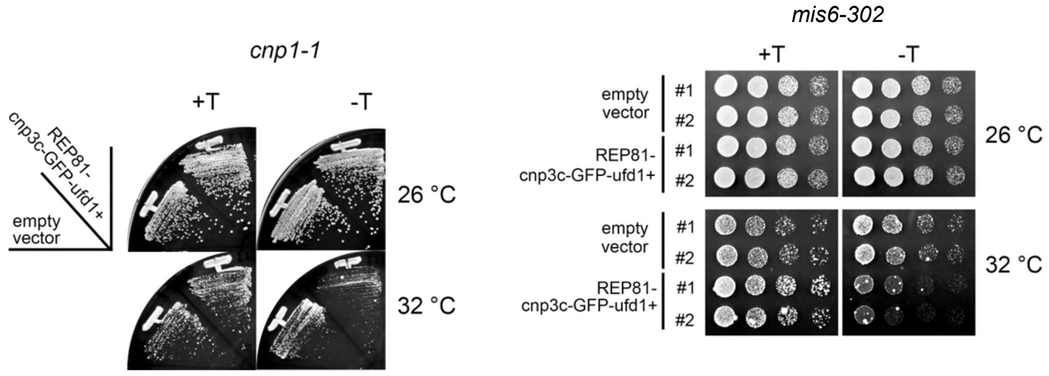

Fig. S3. Artificial enrichment of Ufd1 at the centromere enhances the thermosensitivity of *cnp1-1* and *mis6-302* mutants.

Mutant strains were transformed with pREP81-cnp3c-GFP-Ufd1. *cnp1-1* and *mis6-302* mutants are known to have reduced centromeric CENP-A loading under restrictive temperature conditions (Takahashi et al., 2000). Ectopically expressed cnp3c-GFP-Ufd1 fusion protein resulted in increased thermosensitivity of *cnp1-1* and *mis6-302*.

Biology Open • Supplementary information

Table S1. *S. pombe* strains used in this study

Strain	Genotype	Source
**SP6**	*h^{-} leu1-32*	Matsumoto *et al.*, (2002) (Matsumoto et al., 2002)
**TK28**	*h^{-} lys1::nmt1-GFP-cnp1-lys1^{+} leu1-32*	Kitagawa *et al.*, (2014)
**AW1**	*h^{-} lys1::nmt1-GFP-cnp1-lys1^{+} leu1-32 ufd1-73*	This study
**YKK1114**	*h^{-} lys1::nmt1-GFP-cnp1-lys1^{+} leu1-32 cdc48-353*	This study
**YKK1143**	*h^{-} leu1::nmt1-GFP-h3-leu1^{+}*	This study
**YKK1142**	*h^{-} leu1::nmt1-GFP-h3-leu1^{+} ufd1-73*	This study
**YKK1151**	*h^{-} leu1::nmt1-GFP-h3-leu1^{+} cdc48-353*	This study
**MS122**	*h^{-} lys1::nmt1-GFP-cnp1-lys1^{+} sad1-mCherry::kan^{R} leu1-32*	This study
**YKK1126**	*h^{-} lys1::nmt1-GFP-cnp1-lys1^{+} sad1-mCherry::kan^{R} leu1-32 ufd1-73*	This study
**YKK1124**	*h^{-} lys1::nmt1-GFP-cnp1-lys1^{+} sad1-mCherry::kan^{R} leu1-32 cdc48-353*	This study
**YKK1129**	*h^{-} GFP-cnp1::hph^{R} sad1-mCherry::kan^{R} leu1-32*	This study
**YKK1131**	*h^{-} GFP-cnp1::hph^{R} sad1-mCherry::kan^{R} leu1-32 ufd1-73*	This study
**YKK1125**	*h^{-} GFP-cnp1::hph^{R} sad1-mCherry::kan^{R} leu1-32 cdc48-353*	This study
**YKK1269**	*h^{-} cnt1/TM(NcoI)-ura4^{+} ura4-DS/E GFP-cnp1::hph^{R} leu1-32*	This study
**YKK1250**	*h^{-} cnt1/TM(NcoI)-ura4^{+} ura4-DS/E GFP-cnp1::hph^{R} leu1-32 urd1-73*	This study
**YKK1252**	*h^{-} cnt1/TM(NcoI)-ura4^{+} ura4-DS/E GFP-cnp1::hph leu1-32 cdc48-353*	This study
**YKK1241**	*h^{-} cdc48-GFP::kan^{R} ura4-D18 leu1-32 [pAU-ufd1-RFP]*	This study
**YKK1242**	*h^{-} GFP-cnp1::hph^{R} ura4-D18 leu1-32 [pAU-ufd1-RFP]*	This study
**AW3**	*h^{-} ufd1-GFP-ura4^{+} ura4-D18 leu1-32*	This study
**YKK1196**	*h^{-} ufd1-GFP-ura4^{+} ura4-D18 leu1-32 cdc48-353*	This study
**FY17243**	*h^{-} cdc48-GFP::kan^{R} leu1-32*	YGRC/NBRP
**YKK1197**	*h^{-} cdc48-GFP::kan^{R} leu1-32 ufd1-73*	This study
**YKK1430**	*h^{-} lys1::nmt1-GFP-cnp1-lys1^{+} swi6-tdTomato::hph^{R} leu1-32 ufd1-73 ade6-M210*	This study
**YKK1158**	*h^{-} cnp3-tdTomato::kan^{R} ura4-D18 leu1-32 [pREP81-cnp3c-GFP-ufd1]*	This study

Biology Open • Supplementary information

**YKK1415**	*h^- cdc48-GFP::kan^R cnp3-tdTomato::kan^R ura4-D18 leu1-32 [pREP81-cnp3c-GFP-ufd1]*	This study
**NK91**	*h+ ufd1-GFP-$ura4^+$ ch16-imr3L<<tetO-$ura4^+$ ade6-M210 leu1-32 ura4-D18 [pREP41-npl4-mCherry]*	This study
**YKK1511**	*h^- leu1-32 [pREP81-cnp3c-GFP-ufd1/pREP41-npl4-mCherry]*	This study
**YKK1512**	*h^- leu1-32 [pREP81-cnp3c-GFP-ufd1/pREP41-mCherry]*	This study
**NK5**	*h^- GFP-cnp1::hph ch16-imr3L<<tetO-$ura4^+$ ade6-M210 leu1-32 ura4-D18 [pREP41-tetR-mCherry]*	This study
**NK6**	*h^- GFP-cnp1::hph^R ch16-imr3L<<tetO-$ura4^+$ ade6-M210 leu1-32 ura4-D18 [pREP41-tetR-npl4-mCherry]*	This study
**NK19**	*h^- cdc48-GFP::kan^R ch16-imr3L<<tetO-$ura4^+$ ade6-M210 leu1-32 ura4-D18 [pREP41-tetR-mCherry]*	This study
**NK20**	*h^- cdc48-GFP::kan^R ch16-imr3L<<tetO-$ura4^+$ ade6-M210 leu1-32 ura4-D18 [pREP41-tetR-npl4-mCherry]*	This study
**AW48**	*h^- ufd1-73-GFP-$ura4^+$ ura4-D18 leu1-32*	This study
**MS548**	*h^- GFP-cnp1::hph^R cnp3-mCherry-leu1+ leu1-32*	Suma *et al.*, (2018)
**YKK1477**	*h^- GFP-cnp1::hph^R cnp3-mCherry-leu1+ leu1-32 ufd1-73*	This study
**YKK1483**	*h^- GFP-cnp1::hph^R cnp3-mCherry-leu1+ leu1-32 cdc48-353*	This study
**FY336**	*h^- leu1-32 ade6-M210 ura4-DS/E cnt1/TM(NcoI)-$ura4^+$*	Lab stock
**YKK1139**	*h^- leu1-32 ade6-M210 ura4-DS/E cnt1/TM(NcoI)-$ura4^+$ ufd1-73*	This study
**YKK1141**	*h^- leu1-32 ade6-M210 ura4-DS/E cnt1/TM(NcoI)-$ura4^+$ cdc48-353*	This study
**MT504**	*h^- leu1-32 ade6-M210 ura4-DS/E cnt1/TM(NcoI)-$ura4^+$ mis6-302*	Lab stock
**YKK1182**	*h^- leu1-32 ura4-D18 cnp1::$ura4^+$ cnp1-1:$lys1^+$ [pREP81-cnp3c-GFP-ufd1]*	This study
**YKK1183**	*h^- leu1-32 ura4-D18 cnp1::$ura4^+$ cnp1-1:$lys1^+$ [pREP41]*	This study
**YKK1184**	*h^- leu1-32 mis6-302 [pREP81-cnp3c-GFP-ufd1]*	This study
**YKK1185**	*h^- leu1-32 mis6-302 [pREP41]*	This study

Table S2. PCR primer for Chip assay and plasmids used in this study

cnt1 forward	Kitagawa et al., 2014
cnt1 reverse	Kitagawa et al., 2014
imr1 forward	Takayama et al., 2008 (Takayama et al., 2008)
imr1 reverse	Takayama et al., 2008
dg1 forward	Kitagawa et al., 2014
dg1 reverse	Kitagawa et al., 2014
18S *forward*	Kitagawa et al., 2014
18S *reverse*	Kitagawa et al., 2014
act1 forward	Takayama et al., 2008
act1 reverse	Takayama et al., 2008
ura4 forward	5'-TACCTTTGGGACGTGGTCTC-3'
ura4 reverse	5'-CCCGTCTCCTTTAACATCCA-3'
subtelomere forward	Hayashi et al., 2009 (Hayashi et al., 2009)
subtelomere reverse	Hayashi et al., 2009
pYKK-1	pREP81-cnp3c-GFP-ufd1
pYKK-3	pREP41-cnp3c-ufd1
pYKK-5	pREP41-ufd1
pYKK-6	pREP41-npl4-mCherry
pYKK-7	pAU-ufd1-RFP
pYKK-8	pREP41-tetR-mCherry
pYKK-9	pREP41-tetR-ufd1-mCherry

Biology Open • Supplementary information

References

Gietz, D., St Jean, A., Woods, R. A. and Schiestl, R. H. (1992). Improved method for high efficiency transformation of intact yeast cells. *Nucleic Acids Res* **20**, 1425.

Hayashi, A., Ding, D. Q., Tsutsumi, C., Chikashige, Y., Masuda, H., Haraguchi, T. and Hiraoka, Y. (2009). Localization of gene products using a chromosomally tagged GFP-fusion library in the fission yeast Schizosaccharomyces pombe. *Genes Cells* **14**, 217-25.

Kitagawa, T., Ishii, K., Takeda, K. and Matsumoto, T. (2014). The 19S proteasome subunit Rpt3 regulates distribution of CENP-A by associating with centromeric chromatin. *Nat Commun* **5**, 3597.

Masai, H., Miyake, T. and Arai, K. (1995). hsk1+, a Schizosaccharomyces pombe gene related to Saccharomyces cerevisiae CDC7, is required for chromosomal replication. *EMBO J* **14**, 3094-104.

Matsumoto, S., Bandyopadhyay, A., Kwiatkowski, D. J., Maitra, U. and Matsumoto, T. (2002). Role of the Tsc1-Tsc2 complex in signaling and transport across the cell membrane in the fission yeast Schizosaccharomyces pombe. *Genetics* **161**, 1053-63.

Moreno, S., Klar, A. and Nurse, P. (1991). Molecular genetic analysis of fission yeast Schizosaccharomyces pombe. *Methods Enzymol* **194**, 795-823.

Okazaki, K., Okazaki, N., Kume, K., Jinno, S., Tanaka, K. and Okayama, H. (1990). High-frequency transformation method and library transducing vectors for cloning mammalian cDNAs by trans-complementation of Schizosaccharomyces pombe. *Nucleic Acids Res* **18**, 6485-9.

Pidoux, A. L., Richardson, W. and Allshire, R. C. (2003). Sim4: a novel fission yeast kinetochore protein required for centromeric silencing and chromosome segregation. *J Cell Biol* **161**, 295-307.

Takahashi, K., Chen, E. S. and Yanagida, M. (2000). Requirement of Mis6 centromere connector for localizing a CENP-A-like protein in fission yeast. *Science* **288**, 2215-9.

Takayama, Y., Sato, H., Saitoh, S., Ogiyama, Y., Masuda, F. and Takahashi, K. (2008). Biphasic incorporation of centromeric histone CENP-A in fission yeast. *Mol Biol Cell* **19**, 682-90.
